# Smoke-Free Law Associated With Higher-Than-Expected Taxable Retail Sales for Bars and Taverns in Washington State

**Published:** 2010-06-15

**Authors:** Myde Boles, Julia Dilley, Julie E. Maher, Michael J. Boysun, Terry Reid

**Affiliations:** Program Design and Evaluation Services, Multnomah County Health Department and Oregon Public Health Division; Multnomah County Health Department and Oregon Public Health Division, Portland, Oregon; Multnomah County Health Department and Oregon Public Health Division, Portland, Oregon; Washington State Department of Health, Portland, Oregon; Washington State Department of Health, Portland, Oregon

## Abstract

**Introduction:**

Continued progress in implementing smoke-free laws throughout the United States would benefit from documenting positive economic effects, particularly for the hospitality industry. This study describes changes in sales revenue in bars and taverns since December 2005, when a statewide smoke-free law in Washington State went into effect.

**Methods:**

Using 24 quarters of inflation-adjusted taxable retail sales data from 2002 through 2007, we fitted a regression model to estimate the effect of the smoke-free law on sales revenue, controlling for seasonality and other economic factors.

**Results:**

We found no immediate change in bar revenues in the first quarter of 2006, but taxable retail sales grew significantly through the fourth quarter of 2007. In the 2 years after the smoke-free law was implemented, sales revenues were $105.5 million higher than expected for bars and taverns in Washington State.

**Conclusion:**

The higher-than-expected revenue from taxable sales in bars and taverns after the implementation of smoke-free laws in Washington State provided extra funds to the state general fund. Potential increases in revenue in other jurisdictions that implement smoke-free indoor air policies could provide funds to benefit residents of those jurisdictions.

## Introduction

Approximately one-half of the US population lives in a jurisdiction with some combination of smoke-free workplaces, restaurants, or bars. A total of 31 states, along with Puerto Rico and Washington, DC, have laws in effect that require 100% smoke-free workplaces, restaurants, or bars (1). During the past 15 years, numerous studies on the economic effect of smoke-free laws on the hospitality industry (restaurants, bars, hotels), using objective data and rigorous scientific methods, have been published in the peer-reviewed scientific literature (2-17). Most of these studies have provided clear scientific evidence that there is no deleterious economic effect from smoke-free laws (5-12,14,17); several studies found positive effects (13,15,16), and none showed a negative effect on restaurants and bars.

Of particular interest are studies that have demonstrated the positive economic effect of smoke-free laws on bars (13,15). Tax receipts generated by higher-than-expected taxable sales revenues in bars and taverns can be used in numerous ways by state and local jurisdictions, including antismoking and other public health programs. Studies showing a positive effect provide a financial reason to adopt smoke-free policies, rather than reasoning based on public health benefit alone.

Continued progress in passing and implementing smoke-free laws throughout the United States would benefit from additional demonstration of not only neutral economic effects but especially the positive economic effects, given the tobacco industry's efforts to prevent such laws (2). This is particularly true for the hospitality industry. No studies have quantified the expected financial benefit to state or local jurisdictions of additional taxable sales revenue when smoke-free laws increase sales in bars. The passage of a statewide smoke-free workplace law in Washington State provides a natural experiment to evaluate the economic effect on bars and taverns by using taxable retail sales as objective data to measure outcomes. Specifically, in November 2005, voters in Washington approved Initiative 901, which prohibited smoking in all public places and places of employment. The law went into effect in December 2005. Before that time, bars and taverns were exempted from the state's 1994 smoke-free indoor air law. This report presents the results of an analysis of quarterly sales data from 2002 through 2007 and describes changes in sales revenue in bars and taverns after the December 2005 statewide smoke-free law went into effect.

## Methods

Taxable retail sales (TRS) data were obtained from the Washington State Department of Revenue from the first quarter of 2002 through the fourth quarter of 2007 for bars and taverns (North American Industry Classification System classification 7224 [18]). TRS are sales of tangible personal property and certain services for which a business must collect and remit retail sales tax to the State of Washington. For bars and taverns, TRS represent revenues generated primarily from the sale of food and drink.

Using 24 quarters of inflation-adjusted TRS data from 2002 through 2007, we fit a regression model to estimate the effect of the smoke-free law on sales revenue, controlling for seasonality and other economic factors. Our approach was consistent with other studies of smoke-free laws and restaurant and bar revenues (13,14,17). Specifically, our model took into account autocorrelated error terms by using a first-order autoregressive model with Newey-West standard errors in Stata version 9.2 (StataCorp LP, College Station, Texas). The dependent variable in the regression model was the natural logarithm of inflation-adjusted TRS for bars and taverns. TRS was log-transformed to achieve homogeneity in the error structure because of large TRS values. To express the effect of the smoke-free law in terms of a relative percentage change, we reverse-transformed the coefficients. The model for TRS for bars and taverns was expressed as follows:

ln(TRS_bar)_*i*_ = b_0_ + b_1_SFL_*i*_ + b_2_Q2_*i*_ + b_3_Q3_*i*_ + b_4_Q4*_i_* + b_5_t*_i_* + b_6_SFL_*i*_ t_*i*_+ b_7_UNEMP_*i*_ + b_8_lnPOP_*i*_ + b_9_lnINC_*i*_ + e_*i*_

The subscript *i* denoted time period. SFL was an indicator variable with values of zero in the period before the smoke-free law and values of 1 in the period after the smoke-free law (ie, the first quarter of 2006 through the fourth quarter of 2007). Q2, Q3, and Q4 were quarterly indicator variables to control for seasonal variation, and t_*i*_ was a centered quarterly time trend variable with a value of 0 at the first quarter of 2006. SFL_*i*_ t_*i*_ was the interaction between the smoke-free law indicator and the time trend variable to assess whether the trend in revenue was different after the smoke-free law was implemented. To control for secular economic trends, we included in the equation a seasonally adjusted unemployment variable (UNEMP) obtained from the Washington State Employment Security Department (http://www.workforceexplorer.com). We also included the natural log of Washington state population (POP) and the natural log of Washington state personal income (INC) obtained from the Washington State Economic and Revenue Forecast Council (19) as variables expected to be positively related to sales in bars and taverns. We adjusted revenue and income data for inflation by using the Consumer Price Index for urban consumers in Western states, with December 2007 as the base month (20). The model was tested for multicollinearity in Stata by using the remove collinear procedure, which removes variables with a high variance inflation factor. The Washington State population variable was removed in this procedure. The effect of the smoke-free law was further assessed by comparing the predicted TRS for bars and taverns (from the model) after the implementation of the smoke-free law to the predicted TRS for bars and taverns if there had been no smoke-free law.

## Results

The results of the regression model are in the Table. Because the dependent variable was log-transformed, the time trend variable represented the quarterly TRS compounded growth rate, and the reverse-transformed estimated coefficient for the main effect was interpreted as the quarterly percentage change in TRS before the smoke-free law went into effect. There was no significant change in TRS in the first quarter after the smoke-free law was implemented (*P* = .12). However, the interaction term indicated a significantly more positive trend over time of a 5% increase in revenue each quarter after the smoke-free law went into effect, even after controlling for seasonality and other secular economic trends that could influence the sales in bars and taverns.

At the fourth quarter of 2007, the estimated smoke-free law effect was 0.30 (95% confidence interval, 0.23-0.37) or reverse-transformed value of 1.35. That is, TRS was 35% higher with the smoke-free law than it was projected to be without it. This value was obtained by adding the estimated coefficient for the smoke-free law (−0.05) to 7 times (indicating 7 quarters after the smoke-free law went into effect) the estimated coefficient for the interaction term (7 x 0.05).

The Figure compares the predicted values of TRS for bars and taverns with and without the smoke-free law and shows the divergence of the 2 trend lines over all quarters after the smoke-free law went into effect. The difference calculated from this comparison quantified the quarterly additive increase in bar revenue through the fourth quarter of 2007. The estimated net gain in bar revenue for the 2-year period immediately after implementation of the smoke-free law was $105.5 million.

**Figure.**
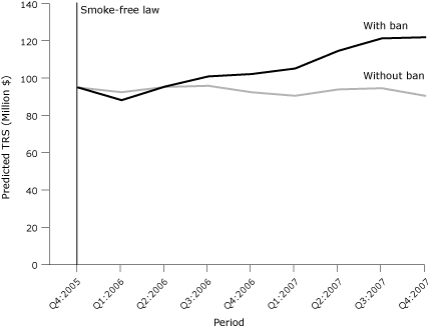
Predicted values from a regression model predicting taxable retail sales (TRS) in bars and taverns in Washington State after the implementation of a smoke-free law, from the fourth quarter of 2005 (Q4:2005) through the fourth quarter of 2007 (Q4:2007). Values are adjusted for seasonality, unemployment, and personal income from 2002 through 2007 (data before Q4:2005 are not shown).

**Table:** 

**Period**	**Predicted TRS (Million $)**	

	**With Ban**	**Without Ban**
Q4:2005	94.66	94.66
Q1:2006	87.81	92.12
Q2:2006	95.17	94.97
Q3:2006	100.68	95.59
Q4:2006	101.96	92.08
Q1:2007	104.96	90.18
Q2:2007	114.56	93.64
Q3:2007	121.27	94.29
Q4:2007	121.87	90.15

## Discussion

The findings in this study demonstrate the positive economic effect quantified in the additional taxable sales revenue in bars and taverns that followed a statewide smoke-free law implemented in Washington State in December 2006. Despite no immediate change in bar and tavern revenues during the first quarter of 2006, taxable retail sales grew significantly through the fourth quarter of 2007. Our analysis suggests that the statewide smoke-free law was associated with higher revenues than would have been expected had the smoke-free law not been in effect.

Our results suggest that not only was bar and tavern revenue increased after a smoke-free indoor air law was passed but also that those increases were large. Because most other studies of smoke-free laws and the hospitality industry have reported no economic effect (5-12,14,17), our unexpected results may be attributed to strong popular support for clean indoor air in Washington State. Voters overwhelmingly approved Initiative 901, imposing one of the strictest smoke-free laws in the nation, clearly indicating a strong preference for clean indoor air in all public places, including bars.

The estimated net gain in Washington of more than $105 million during the 2 years after implementation of the smoke-free law translates into a $21 per capita increase ($105.5 million divided by 5 million adult population) in spending. In Washington State, tax receipts generated from 6.5% sales tax and 0.5% business and occupation tax on taxable retail sales at bars go to the state's general fund. That translates to approximately $7.4 million, which can be used for programs to benefit residents of the state. Although many factors can influence revenue for bars and taverns, other states may expect to see similar increases in revenue when passing smoke-free indoor air laws. Further, our findings underestimate the total gain in net income to bars, as costs associated with cleaning may be reduced when smoking is no longer allowed indoors.

Revenue gains may be an effect both of new nonsmoking patrons going to bars and existing smoking bar patrons continuing to go to bars. Population-based surveys of dining and drinking behaviors indicate that few smokers change their behavior, while nonsmokers are more likely to patronize the newly smoke-free venues (2,21). Because smokers have few alternatives to bars once they are smoke free, empiric evidence in California shows that the 1998 smoke-free bar law increased bar revenues by attracting more nonsmokers, while having little effect on existing smokers (13). Our long-term data suggest that revenues eventually become higher than prior levels. Therefore, we may expect that future years will continue to be more profitable than they would have been without the smoke-free law.

This study has limitations. First, because the study examined aggregate TRS for all bars and taverns in Washington State, a few individual establishments may have been negatively affected by the smoke-free law. However, the decrease in revenue for these establishments was more than offset by the increase in revenue overall and supports our conclusion about the positive state-level economic effect. Second, the number of establishments increased from 1,020 in the first quarter of 2006 to 1,117 in the fourth quarter of 2007. A portion of the increase in TRS could be attributed to the growth in the number of establishments rather than increased patronage at existing locations, but the expansion is an indicator of a thriving sector of the economy that was not hurt by the smoke-free law. Third, bars may have increased prices to offset any drop in sales that could have been caused by the smoke-free law, and the growth in TRS may have been because of higher prices. Although we did not analyze price data, if the smoke-free law had lowered demand for bars, then economic theory suggests that prices would have fallen rather than risen. Finally, because our study was conducted in a state where the smoke-free law was passed by a ballot initiative, our results may not be generalizable to other states where laws are passed without popular support.

Strategies used in tobacco use prevention and control programs, including strong smoke-free indoor air policies, are a good return on investment for other reasons as well. In particular, strong smoke-free indoor air policies immediately reduce the incidence of heart attack hospitalizations (22-25). Future research on the economic effect of smoke-free laws should focus on the economic benefits of reduced hospitalizations and other associated medical care costs.

Because many states have yet to pass comprehensive clean indoor air laws, this study can help to ameliorate concerns that such laws will have an adverse effect on bars. Our findings provide additional reasons for states to adopt strong smoke-free indoor air policies for all workplaces, including bars. In addition to the obvious public health benefits and possible decreases in health care costs, smoke-free indoor air policies may increase revenues for bar and tavern businesses, which may, in turn, provide an economic return to state and local jurisdictions in the form of additional tax receipts that may be used to benefit residents of those jurisdictions.

## Figures and Tables

**Table.** Regression Model Results^a^ for Taxable Retail Sales (TRS) in Bars and Taverns, Washington State, 2002-2007

**Independent Variable**	Estimated Coefficient	Multiplicative Change^b^	Standard Error	*P* Value
Smoke-free law (SFL)	−0.05	0.95	0.03	.12
Q2	0.05	1.05	0.02	.01
Q3	0.08	1.08	0.02	<.001
Q4	0.04	1.04	0.02	.03
Time (quarters)^c^	−0.01	0.99	0.00	<.001
Time × SFL interaction	0.05	1.05	0.01	<.001

a First-order autoregressive model with Newey-West standard errors with ln(TRS) as outcome. The model included all the independent variables in the table, unemployment (*P* < .001), and personal income (*P* = .89). Model *R^2^* was 0.90 from non-autoregressive regression model.

b Obtained by reverse-transforming (ie, exponentiating) the estimated coefficients.

c Time centered with a value of zero at first quarter of 2006.
